# The need to improve antimicrobial susceptibility testing capacity in Ugandan health facilities: insights from a surveillance primer

**DOI:** 10.1186/s13756-022-01072-4

**Published:** 2022-02-03

**Authors:** Duku Chaplain, Butti Ben Asutaku, Muhammad Mona, Douglas Bulafu, Dickson Aruhomukama

**Affiliations:** 1 Clinical Microbiology Laboratory, Mulago National Referral Hospital, Kampala, Uganda; 2 Department of Disease Control and Environmental Health, School of Public Health, College of Health Sciences, Makerere University, Kampala, Uganda; 3 Department of Immunology and Molecular Biology, School of Biomedical Sciences, College of Health Sciences, Makerere University, P.O. Box 7072, Kampala, Uganda; 4 Department of Medical Microbiology, School of Biomedical Sciences, College of Health Sciences, Makerere University, Kampala, Uganda

**Keywords:** Antimicrobial resistance, Surveillance, Microbiology labs, Mulago Hospital, Uganda

## Abstract

**Background:**

Lab-based surveillance (LBS) of antimicrobial resistance (AMR) is not systematically implemented in Uganda. We aimed to identify the gaps in establishing regular LBS of AMR in Uganda.

**Methods:**

This was a retrospective records review. It was done at Mulago Hospital (MH) Microbiology Lab (MHL). It analyzed lab records on bacteria and their antimicrobial susceptibility profiles (ASPs) over 6 months. Since MH is the national referral hospital, we hypothesized that (1) MHL is the best resourced and that any limitations seen here are amplified in labs at regional referral hospitals (RRHs) and health centers (HCs); (2) data from MHL on LBS mirrors that from labs at RRHs and HCs; (3) the state of records from MHL show lab performance and the presence or absence of standard operating procedures (SOPs), as would be the case at RRHs and HCs.

**Results:**

The lab got 1760 samples over the six months. The most common sample was urine (659, 37.4%). From the 1760 samples, data on 478 bacterial isolates were available. Urine gave the most isolates (159, 33.3%). Most of the isolates were gram-negative (267, 55.9%). *Escherichia coli* (100, 37%) was the most common gram-negative (of the Enterobacteriaceae). *Pseudomonas aeruginosa* (17, 6%) was the most common gram-negative (of the non-Enterobacteriaceae). The gram-negative bacteria were highly resistant to β-lactams. These were highly sensitive to carbapenems. The *Staphylococcus aureus* were highly resistant to β-lactams. The gram-positive bacteria were highly sensitive to vancomycin. ASPs for all bacterial categories were incomplete.

**Conclusions:**

The findings from MHL suggest that there is a need to improve antimicrobial susceptibility testing capacity. They also suggest that the situation at RRHs and HCs around the country could be worse. So, there is a need to extend the political will, which already exists, into operational and implementation action.

## Introduction

Antimicrobial resistance (AMR) continues to be a public health threat of global interest [1]. Predictions show that about 10 million people will die yearly from infections caused by AMR bacteria till 2050 [2]. But, this remains subject to how the globe reacts to reduce the burden of AMR [3, 4]. Africa is one of the regions likely to contribute the highest numbers to these global deaths [2].

Surveillance is a vital tool to inform and monitor local, national, and global strategies towards reducing the burden of AMR [5]. Lab-based surveillance (LBS) involves AST, data management, analysis, and reporting [5, 6]. The other part of LBS is the development of SOPs [5, 6].

A more practical way of doing regular LBS would be to establish a standardized programme from top–bottom. The programme would ensure that the participating labs access the SOPs and supplies needed to realize the plan. Like other countries, Uganda has established such a programme, the AMR National Action Plan (AMR NAP) [7]. The AMR NAP outlines surveillance as one of its strategic aims [7]. Despite this, regular LBS is not systematically implemented in Uganda [8, 9]. The country collects and uses data from short-term researcher-driven studies for surveillance [8, 9].

To identify the gaps in establishing regular LBS in Uganda, we focused our study on the lab (MHL). Since MH is the national referral hospital, we hypothesized that (1) MHL is the best resourced and that any limitations seen here are amplified in labs at regional referral hospitals (RRHs) and health centers (HCs); (2) data from MHL on LBS mirrors that from labs at RRHs and HCs; (3) the state of records from MHL show lab performance and the presence or absence of SOPs, as would be the case at RRHs and HCs. The study design had a vital impact on the drafting of the hypotheses.

We retrospectively analyzed records for the prevalence of common bacteria from patient samples at MHL. We also analyzed the antimicrobial susceptibility profiles (ASPs) of the bacteria.

## Methods

### Design, site and settings

This was a retrospective records review. It was carried out at MHL. Mulago Hospital (MH) is the national referral hospital in Uganda. It gives free health care services that include lab services. Its microbiology lab (MHL) receives and tests samples that are routinely taken from patients. The choice to get samples from patients relies solely on clinicians and no guidelines exist. Also, there is no dedicated sample transportation service provider and sample transportation from the points of collection (hospital’s wards) to MHL is done by the patients or their caretakers. However, it is also not mandatory that the samples be tested at MHL. So, even though MHL receives and tests the largest number of the collected samples, some samples never reach this lab. These samples are tested in private facilities. The decision to test at these facilities relies on the patient or their caretaker. This partly highlights the lack of routine and standardized processes for AST of specimens. Testing in MHL includes culture for both gram-negative and -positive bacteria that is done using solid, semi-solid, and liquid media. Culture also includes the identification of the bacteria using urged biochemicals. AST for the bacteria is done using the Kirby Bauer disk diffusion method. The performance of AST is done as per the clinical and laboratory standards guidelines (CLSI). No electronic data management, analysis, and reporting system exist in MHL. The generated data is managed and analyzed manually. Also, results are reported manually. The data is stored in paper form. No records of any staff training were available to the study.

### Study description, data management and analysis

The study retrieved the lab paper-based records between November 2020 and April 2021. Data on the identity (ID) of the bacterial isolates and their ASPs was extracted from the lab paper-based records, entered, and cleaned using Microsoft Excel 2016 (Microsoft Corporation, Redmond, WA). Descriptive analysis of the data was still done using Microsoft Excel 2016 (Microsoft Corporation, Redmond, WA). The data was presented in percentages and frequencies. No data beyond bacterial IDs and their ASPs was available and so could not be analyzed by the study.

## Results

### Bacteria isolated from samples

A total of 478 isolates had been obtained from 1760 samples. A total of 478 isolates had been got from 1760 samples. The 1760 samples were urine (659, 37.4%), swabs (439, 24.9%), blood (389, 22.1%), cerebrospinal fluid (164, 9.3%), aspirates (67, 3.8%), and stool (42, 2.5%). The 478 isolates had been got from the samples as follows; urine (159, 33.3%), swabs (265, 55.4%), blood (40, 8.4%), cerebrospinal fluid (6, 1.3%), and aspirates (8, 1.8%). The most prevalent of the Enterobacteriaceae was *E. coli* (37%). *P. aeruginosa* (6%) was the most prevalent of the non-Enterobacteriaceae. Unidentified gram-negative bacteria (UGNB) were 16% of the gram-negative bacteria isolates. *S. aureus* (83%) was the most prevalent gram-positive bacteria (Table 1). Data on co-infections (presence of greater than one species in a sample) was not available and was not captured.

**Table 1:** Bacteria isolated from the samples

Isolated bacteria (N = 478)			
Gram-negative bacteria (n = 267, 55.9%)	f (%)	Gram-positive bacteria (n = 211, 44.1%)	f (%)
^*#*^*E. coli*	100 (37)	^**^CNS	20 (10)
^*#*^*C. freundii*	64 (24)	*S. aureus*	176 (83)
^*#*^*Proteus spp*	13 (5)	*S. viridians*	4 (2)
^*#*^*M. morganii*	1 (0.4)	*S. pyogenes*	3 (1)
^***#***^*S. marcescens*	1 (0.4)	*S. pneumoniae*	2 (1)
^*#*^*K. pneumoniae*	26 (10)	*Enterococcus spp*	6 (3)
^*#*^*Enterobacter spp*	1 (0.4)		
^+^*P. aeruginosa*	17 (6)		
^*^Others	44 (16)		

*Others-UGNB, ^#^Enterobacteriaceae, ^+^non-Enterobacteriaceae, **Coagulase negative staphylococcus

### Antimicrobial susceptibility profiles of selected gram-positive bacteria

The *S. aureus* were highly resistant to among others ampicillin (86.8%). They were highly sensitive to among others vancomycin (86.3%). The *Streptococcus spp* were highly resistant to among others ampicillin (100%). They were highly sensitive to among others vancomycin (100%). The *Enterococcus spp* were highly sensitive to among others tetracycline (100%). They were highly resistant to among others ciprofloxacin (100%). In all cases, only a few bacteria had been tested for particular antimicrobials. This is because the antimicrobials were either not available or not recommended by the CLSI (Table 2).

**Table 2:** Antimicrobial susceptibility profiles of the most common gram-positive bacteria

Antimicrobials	*S. aureus*		*S. pneumoniae*		*S. pyogenes*		*Enterococcus spp*		*S. viridians*	
	R n (%)	S n (%)	R n (%)	S n (%)	R n (%)	S n (%)	R n (%)	S n (%)	R n (%)	S n (%)
AMP	46 (86.8)	7 (13.2)	1 (50)	1 (50)	1 (100)	0 (0)	3 (50)	3 (50)	1 (100)	0 (0)
A	1 (100)	0 (0)	c	c	a	a	c	c	a	a
FOX	17 (32.7)	33 (63.5)	c	c	c	c	c	c	c	c
SXT	39 (66.1)	19 (32.2)	1 (100)	0 (0)	c	c	c	c	c	c
CIP	21 (52.5)	18 (45)	c	c	c	c	2 (100)	0 (0)	c	c
CN	51 (52.6)	46 (46.9)	c	c	c	c	c	c	1 (100)	0 (0)
P	a	a	a	a	a	a	a	a	a	a
VAN	13 (13.7)	82 (86.3)	0 (0)	1 (100)	0 (0)	1 (100)	1 (33.3)	2 (66.7)	0 (0)	1 (100)
AMC	c	c	1 (50)	1 (50)	c	c	c	c	c	c
C	28 (42.4)	38 (57.6)	1 (100)	0 (0)	a	a	a	a	a	a
DA	9 (19.1)	38 (80.9)	a	a	a	a	c	c	a	a
E	c	c	a	a	1 (100)	0 (0)	1 (50)	1 (50)	1 (100)	0 (0)
N	1 (16.7)	5 (83.3)	c	c	c	c	1 (100)	0 (0)	c	c
CTX	c	c	a	a	a	a	c	c	a	a
IMP	c	c	a	a	a	a	c	c	a	a
CRO	c	c	a	a	a	a	c	c	a	a
TE	12 (80)	2 (13.3)	a	a	a	a	0 (0)	3 (100)	a	a
CXM	c	c	a	a	a	a	c	c	c	c
RIF	3 (16.7)	15 (83.3)	0 (0)	1 (100)	c	c	1 (50)	1 (50)	c	c
AZT	c	c	c	c	c	c	c	c	c	c

R—Resistant, S—Sensitive, %—bacteria screened against a particular antimicrobial, a and c—antimicrobial not screened against the bacteria (due to c—CLSI guidelines or a—total absence or unavailability in the lab), AMP—Ampicillin, A—Azithromycin, FOX—Cefoxitin, CN—Gentamicin, IPM—Imipenem, CIP—Ciprofloxacin, CTX—Cefotaxime, AZT—Aztreonam, AMC—Amoxicillin-clavulanic acid, N—Nitrofurantoin, SXT—Cotrimoxazole, CXM—Cefuroxime, CRO—Ceftriaxone, C—Chloramphenicol, P—Penicillin, VAN—Vancomycin, DA—Clindamycin, E—Erythromycin, TE—Tetracycline, RIF—Rifampicin

### Antimicrobial susceptibility profiles of the most common gram-negative bacteria

The profile showed that *E. coli*, *K. pneumoniae*, *C. freundii*, and UGNB were highly resistant to among others nalidixic acid (100%), ampicillin (100%), and cefotaxime (100%). The profile also showed that *P. aeruginosa* were highly resistant to aztreonam (100%). The profile showed that *E. coli*, *K. pneumoniae*, *C. freundii*, and UGNB were highly sensitive to imipenem (> 93%). On the other hand, the *P. aeruginosa* were highly sensitive to piperacillin (100%) and ciprofloxacin (100%) (Table 3). In all cases, only a few bacteria had been tested for particular antimicrobials. This is because the antimicrobials were either not available or not recommended by the CLSI (Table 3).

**Table 3:** Antimicrobial susceptibility profiles of the most common gram-negative bacteria

Antimicrobials	*E. coli*		*K. pneumoniae*		*C. freundii*		*P. aeruginosa*		UGNB	
	R n (%)	S n (%)	R n (%)	S n (%)	R n (%)	S n (%)	R n (%)	S n (%)	R n (%)	S n (%)
CN	32 (48.5)	33 (50)	10 (66.7)	4 (26.6)	16 (80)	4 (20)	1 (10)	8 (80)	7 (77.8)	1 (11.1)
IMP	0 (0)	77 (100)	0 (0)	20 (100)	2 (50)	2 (50)	1 (7.7)	12 (92.3)	1 (6.7)	14 (93.3)
CAZ	38 (65.5)	1 (29.3)	8 (100)	0 (0)	14 (82.4)	2 (11.7)	1 (10)	9 (90)	4 (44.4)	3 (33.3)
CIP	28 (74.5)	8 (21.6)	7 (58.3)	4 (33.4)	9 (64.3)	4 (28.6)	0 (0)	6 (100)	7 (53.8)	6 (46.2)
CTX	13 (86.7)	2 (13.3)	1 (100)	0 (0)	1 (100)	0 (0)	c	c	6 (100)	0 (0)
AMP	26 (96.3)	1 (3.7)	5 (100)	0 (0)	8 (100)	0 (0)	c	c	14 (100)	0 (0)
AZT	2 (100)	0 (0)	a	a	2 (100)	0 (0)	1 (100)	0 (0)	1 (100)	0 (0)
AMC	18 (27.7)	43 (66.1)	3 (25)	9 (75)	10 (37.1)	15 (55.5)	c	c	4 (33.3)	8 (66.7)
N	5 (10.4)	4 (85.4)	0 (0)	3 (100)	4 (50)	4 (50)	c	c	0 (0)	3 (75)
SXT	32 (91.4)	3 (8.6)	7 (87.5)	1 (12.5)	10 (90.9)	1 (9.1)	c	c	0 (0)	0 (0)
CXM	2 (100)	0 (0)	a	a	6 (100)	0 (0)	c	c	2 (50)	2 (50)
NA	13 (59.1)	7 (31.8)	1 (100)	0 (0)	5 (71.4)	2 (28.6)	c	c	3 (100)	0 (0)
CRO	2 (69)	8 (27.6)	7 (75)	3 (25)	6 (100)	0 (0)	c	c	3 (60)	2 (40)
C	9 (36)	16 (64)	5 (50)	5 (50)	9 (56.3)	7 (43.7)	c	c	1 (100)	0 (0)
AK	0 (0)	2 (66.7)	a	a	a	a	a	a	1 (100)	0 (0)
FOX	5 (83.3)	1 (16.7)	1 (33.3)	2 (66.7)	5 (62.5)	3 (37.5)	c	c	a	a
MEM	0 (0)	3 (100)	a	a	0 (0)	1 (100)	a	a	a	a
TPZ	a	a	a	a	a	a	0 (0)	3 (100)	a	a

R—Resistant, S—Sensitive, %—bacteria screened against a particular antimicrobial, a and c—antimicrobial not screened against the bacteria (due to c—CLSI guidelines or a—total absence or unavailability in the lab), CN—Gentamicin, IPM—Imipenem, CAZ—Ceftazidime, CIP—Ciprofloxacin, CTX—Cefotaxime, AMP—Ampicillin, AZT—Aztreonam, AMC—Amoxicillin-clavulanic acid, N—Nitrofurantoin, SXT—Cotrimoxazole, CXM—Cefuroxime, NA—Nalidixic acid, CRO—Ceftriaxone, C—Chloramphenicol, AK—Amikacin, MEM—Meropenem, FOX—Cefoxitin, MEM—Meropenem, TPZ—Tazobactam piperacillin

## Discussion

This study found a high prevalence of *E. coli, P. aeruginosa*, and *S. aureus*. These findings are similar to those of many short-term researcher-driven studies earlier done at MH, which stated a high prevalence of the same species [9–15]. These species continue to cause the commonest community- and hospital-acquired bacterial infections [9–15]. But, these species could be used as surveillance markers for AMR in this setting as has been shown [16]. Centering LBS of AMR on these species could promote the efficient use of the limited resources for ASP.

Broadly, our ASPs of the different bacterial species were similar to ASPs reported for the same species in many studies earlier done at MH [9–15]. Lab-based surveillance of AMR hinges on the availability of reliable ASP data [6]. Microbiology labs create such data and can drive the surveillance [9, 17]. This finding hence shows the potential of MHL to steer LBS of AMR efforts in Uganda. In spite of this fact, no system is in place to ensure that findings from MHL are always conveyed to the relevant state authorities. This remains one of the gaps in the establishment of LBS of AMR in Uganda.

This study found that all ASP data that was available was incomplete. The lack of complete ASP data is not unique to this study. It has also been reported in some studies earlier done at MH [9, 15]. The lack of complete ASP data could be attributed to the fact that testing for some antimicrobials was never done due to their unavailability, testing was never done at all, and possible loss of data during its manual management. The lack of complete ASP data makes AMR surveillance attempts difficult [6]. In some settings, data loss is prevented by the use of Lab Information Management systems (LIMS). These systems have enabled the shift from paper- to electronic-based record keeping [18]. Electronic data capture platforms allow more reliable data storage and retrieval capabilities [19]. But, to efficiently use LIMS, personnel must be trained on how to use them, tools such as more complex computer hardware must be made ready to host them, and an able team for system maintenance must be in reach to always fix hurdles due to the use of the systems [18]. In spite of these likely challenges, consortia could be established with similar labs to obtain multiuser licenses for LIMS [18], or locally developed, open source LIMS could be used for example the SILAB, Baobab, and WHONET [20–22]. The failure to adopt the use of such LIMS remains one of the gaps to the establishment of LBS of AMR in Uganda. This is because it promotes the loss of ASP data and prevents ease of conveying actionable ASP data to relevant state authorities. The other reasons for the incompleteness of ASP data are discussed later.

This study found that some bacteria had been unidentified (UGNB). In spite of their small numbers, the similarity of their susceptibility profiles to those named makes them important. This also beckons for their IDs to be made known. Reports of unknown bacteria are not unique to this study. This nomenclature (UGNB) has been stated in many studies earlier done in Uganda [13, 23–25]. This suggests the failure of lab staff to ably group bacteria [26, 27]. The failure to correctly group bacteria affects the reliability of ASP and as a result, negatively affects the precision of therapy [28–31]. Due to the fact that this study did not find staff training records, the staff may be untrained or trained but in need of fresh training. Such states of lab staff cannot ensure reliable ASP. Unreliable ASP also affects surveillance efforts. Constant training of lab staff and participation in external competence assessments has been shown to improve staff skills to group bacteria well [32–35]. The use of semi-automated or automated grouping systems has been shown to improve the grouping [36–38]. But, these could be costly to buy, run and are hence impractical for limited-resource settings (LRS) [39–41]. Investing resources towards the Improvement of staff skills can improve their ability to identify bacteria and do reliable ASP. This is most practical option for LRS.

This study found that the number of bacteria that had been tested with specific antimicrobials was way lower than the actual numbers that had been tested. A similar finding has been reported in studies earlier done at MH [9, 15]. Although this finding could have also been due to the reasons mentioned earlier i.e. the fact that testing for some antimicrobials was never done due to their unavailability, testing was never done at all, and possible loss of the testing data during its manual management. But, in this case, the fact that the testing was never done at all and the likelihood of data loss are unlikely. We attribute this finding to the lack of supplies needed to do ASP. The shortage of the supplies needed to do ASP steers the incompleteness and unreliability of ASP data [42–44]. This, in turn, affects therapy, leads to needless expenditures in settings already plagued by resource shortages, advances the thought that lab testing is unhelpful, and sequentially makes data-driven surveillance difficult [42–44]. To ensure the availability of supplies, the use of inventory logs can be made a routine. Inventory logs ensure that supplies are always tracked. So, the efficient use of the logs can enhance financial and supply management to ensure that the needed supplies are in stock regularly.

Despite the existence of the AMR NAP, this study suggests that there have not been corresponding improvements at the hospital level in enhancing AMR surveillance, even at one of the best-resourced hospitals like Mulago. However, as noted earlier, the fact that the plan exists means that there is already political attention. So, there needs to be an extension of the political will, that already exists, to move from having a high-level policy in place (the AMR NAP) to concrete improvements at the hospital and lab level.

The study was unable to obtain complete ASP data sets since they were absent. This limited our analyses and hence could have negatively influenced our discussion of the results. Although this was a study limitation, it was beyond our control. The study also reviewed records that had been collected over a short period. Reviewing records over a long period could have produced better numbers for the profiled bacteria. This could have extended our analyses enabling us to discuss the results adequately. This was a study limitation. The records that were available to the study comprised only the IDs of isolated bacteria and their ASPs. This was a study limitation as it stopped us from reporting on the links of the findings to particular diagnoses or pathologies in the patients. We also did not ascertain whether or not the lab kept inventory logs, this could have affected the way we discussed the results on the availability of supplies. This too was a study limitation.

## Conclusions

This study exhibited the need to boost the capacity of clinical microbiology labs if they are to steer LBS of AMR in Uganda. This can be made possible by ensuring the steady supply of materials needed to prepare samples and do ASP, installing LIMS by which ASP-related data can be archived and shared, and training staff to prepare samples and do ASP continuously. Broadly, the findings from MHL suggest the worse situation in similar labs at RRHs and HCs around Uganda, with fewer available resources. This speaks to the need for a concerted effort to extend the political will, which already exists, to ensure the availability of resources needed to do regular LBS of AMR.

## Data Availability

The data that support the findings of this study are available from the MHL but restrictions apply to the availability of these data, which were used under license for the current study, and so are not publicly available. Data are however available from the authors upon reasonable request and with permission of the in-charge of the MHL.

## Abbreviations

AMR: Antimicrobial resistance
ASPs: Antimicrobial susceptibility profiles
ASP: Antimicrobial susceptibility profiling
AMR NAP: Antimicrobial resistance national action plan
CLSI: Clinical Laboratory Standards Institute
HCs: Health Centers
LBS: Lab-based surveillance
MH: Mulago Hospital
MHL: Mulago Hospital Microbiology Lab
RRHs: Regional Referral Hospitals
SOPs: Standard operating procedures
UGNB: Unidentified gram-negative bacteria
